# In-Vitro Evaluation of Dental Adhesive Bond Strength With Diode Laser Irradiation Before Photopolymerization

**DOI:** 10.7759/cureus.56935

**Published:** 2024-03-26

**Authors:** Srikumar GPV, Vaishali Shukla, Arti A Raut, Megha Ghosh, Mohammed Mustafa, Raneem Suleiman Alofi

**Affiliations:** 1 Department of Conservative Dentistry and Endodontics, Triveni Institute of Dental Sciences, Hospital and Research Centre, Bilaspur, IND; 2 Department of Conservative Dentistry and Endodontics, Triveni Institute of Dental Sciences, Hospital and Research, Bilaspur, IND; 3 Department of Conservative Dentistry and Endodontics, Center for Transdisciplinary Research (CFTR) Saveetha Dental College, Saveetha Institute of Medical and Technical Sciences, Saveetha University, Chennai, IND; 4 Department of Restorative Dental Sciences, College of Dentistry, King Saud University, Riyadh, SAU

**Keywords:** shear bond strength, dentistry, photopolymerization, irradiation, diode-laser, adhesive-dentin interface

## Abstract

Aim: In-vitro evaluation of shear bond strength, mode of failure, and adaptation of fifth-generation (etch-and-rinse), seventh-generation,and eighth-generation self-etch dental adhesives to human dentin with or without diode-laser irradiation before photopolymerization.

Materials and methods: Seventy-two extracted human maxillary premolar teeth were collected. The buccal and lingual surfaces of teeth were grounded until dentin was exposed. Test areas of 4 mm diameter were created on both surfaces of teeth to standardize the area of treatment. The samples were then randomly allocated into three groups (n = 24): Group 1 Adper Single Bond 2 Etch-and-Rinse; Group 2 Tetric-N-Bond Universal Self-Etch; Group 3 Prime and Bond Universal Self-Etch dental adhesives were used. Buccal surfaces (sub-groups ‘a’) of all specimens were irradiated with diode laser before photopolymerization of the adhesive material, and palatal surfaces (sub-groups ‘b’) were directly photopolymerized without prior diode laser irradiation and restored with composite resin. All specimens were thermocycled. Four specimens from each group were then subjected to scanning electron microscopy (SEM) analysis to examine the adaptation of adhesive to dentin, and the remaining 60 specimens were evaluated for shear bond strength tests, modes of failure at the adhesive-dentin interface, and values were recorded, tabulated, and used for data analysis. A one-way ANOVA test and the Student's t-test were used for statistical analysis. A P value ≤ 0.05 was considered statistically significant.

Results: The mean shear bond strength for the groups was: Group 1a (13.96 MPa), 1b (14.95 MPa); Group 2a (10.06 MPa), 2b (10.30 MPa); Group 3a (12.03 MPa), and 3b (10.44 MPa). No statistically significant difference was seen among sub-groups 1a and 3a, 2a and 3a, 2b and 3b as P > 0.05. A significant difference was seen among sub-groups 1b and 3b (P<0.05), 1a and 2a, and 1b and 2b (P<0.01).

Conclusion: Adper Single Bond 2 without diode-laser irradiation before photopolymerization showed the highest shear bond strength, followed by Adper Single Bond 2 irradiated with diode laser before photopolymerization, with the maximum adaptation of dental adhesive to dentin compared to other adhesives used either with or without diode-laser irradiation.

## Introduction

With the advent of the concept of Bonding in operative dentistry, dental adhesion achieved significant importance. Since then, dental composite restorations gained immense popularity and are today considered as ideal tooth-coloured restorative material. Dental adhesion creates an intimate contact between composite restoration and tooth structure, thus preventing marginal gaps, microleakage, and secondary caries and enhancing the color stability and clinical longevity of restorations [1]. While effective adhesion to tooth enamel is achieved with ease, adhesion to dentin poses a great challenge and is partly due to characteristics of dentin, mainly its high organic content, tubular structure, and formation of smear layer immediately after tooth preparation [2]. For effective adhesion, the smear layer can either be removed as in the total-etch technique or modified as in the self-etch technique.

The total-etch technique, also termed the 'etch-and-rinse' approach, was introduced in the early 1990s and involves the etching of enamel and dentin simultaneously using 37% phosphoric acid, followed by rinse and application of a fifth-generation dentin bonding agent consisting of primer and adhesive in one solution. To date, it is considered a ‘gold-standard’ adhesive system [3]. To overcome the adverse effects of over-and under-etching and the multi-step bonding approach in the total-etch technique, the demand for a simplified adhesive approach led to the development of self-etch adhesives. The advantage of using self-etch adhesives is that prior removal of the smear layer and smear plugs is not required, as these adhesives simultaneously etch the tooth surface and also prepare it for adhesion [4]. Self-etch adhesive enables simultaneous etching, priming, and application of bonding, thus simplifying and shortening the adhesion process of adhesion [5].

Tetric N-Bond Universal (Ivoclar Vivadent, Liechtenstein) is a seventh-generation (single-component with both self-etch and etch-and-rinse bonding techniques), universal dental adhesive system. It is a light-cured adhesive composed of Bisphenol A-Glycidyl Methacrylate (Bis-GMA), phosphoric acid ethanol, urethane dimethylacrylate, and a combination of hydrophilic, 2-hydroxyethyl methacrylate, hydrophobic, and 1,10-decandioldimethacrylate (D3MA) allowing Tetric N-Bond Universal to reliably bridge the gap between the hydrophilic tooth substrate and the hydrophobic restorative resin, thus enhancing adhesion with improved bond strength. Nanotechnology in dentistry has led to the development of nanocomposites and nanodental adhesives. Nano‑adhesives are solutions with nano‑sized fillers (silica nanoparticles, barium glass nanoparticles, and other proprietary filler materials) enhancing optimum enamel and dentin bond strength, stress absorption, and longer shelf life [6]. Prime and Bond Universal (Dentsply Sirona, Switzerland) is an eighth-generation universal dental adhesive composed of bi- and multifunctional acrylate as a surface active crosslinker, phosphoric acid-modified acrylate resin as an etchant, adhesion promoter, and primer, a stabilizer to stabilize monomers upon storage, isopropanol as a solvent, and polarity adjustment [7].

Goncalves et al. [8] suggested the use of Neodymium: Yttrium-Aluminium- Garnet (Nd: YAG) laser irradiation of already infiltrated simplified dentin adhesives before their photopolymerization, promoting better adhesion to the tooth with increased bond strength values. However, in our study, we used the diode laser as an alternative, as it provides near-infrared irradiation with parameters similar to those of the Nd: YAG laser and also owing to its higher affordability, portability, smaller size, and weight [9-11].

Hence, this in-vitro study aimed to evaluate the macro-shear bond strength, mode of failure, and adaptation of fifth-generation (etch-and-rinse), universal bonding agents [(Tetric N-Bond Universal (Ivoclar Vivadent, Liechtenstein) and Prime and Bond Universal (Dentsply Sirona, Switzerland) dental adhesives to human dentin with or without diode laser irradiation prior to photopolymerization.

## Materials and methods

An in-vitro study was conducted in the Department of Conservative Dentistry and Endodontics after obtaining the Institutional Ethical Committee clearance certificate, TIDSHRC/IEC/2023/D0021, of the Triveni Institute of Dental Sciences, Hospital and Research Center, Bilaspur, Chhattisgarh, India. Our study sample consisted of 72 human permanent maxillary first and second premolar teeth, extracted either for orthodontic reasons or periodontally compromised. All teeth were examined under a stereomicroscope (Olympus SZ61, Olympus Optical Co., Tokyo, Japan) at 10X magnification to ensure that they were intact and without any caries or non-caries lesions, devoid of restorations, clinically detectable fractures, cracks, or enamel hypoplasia, following strict inclusion criteria. All specimens utilized in our study were within one month of extraction. The collected teeth were cleaned of superficial debris, calculus, and residual tissue tags using ultrasonic instruments. Occupational Safety and Health Administration (OSHA) and Center for Disease Control (CDC) and Prevention recommendations and guidelines were followed during the collection, sterilization, and handling of extracted teeth, and they were stored in 0.5% thymol at room temperature until used to prevent biohazard transmission.

**Figure 1:**
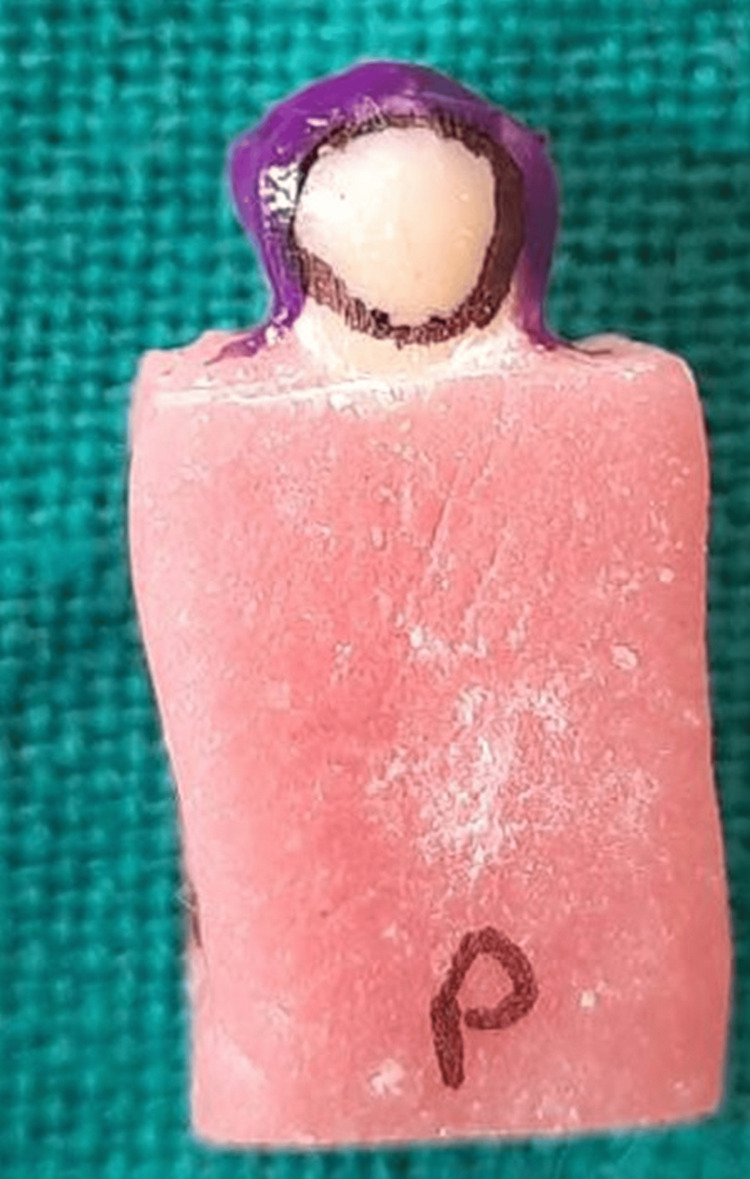
Prepared tooth and prepared test area

The specimens were then individually embedded in self-cure acrylic resin (DPI-RR Cold Cure, Mumbai, India) blocks up to the level of the cementoenamel junction, exposing only the crown portion of the teeth to facilitate ease of handling. The buccal and palatal surfaces of teeth were then grounded using a fine-grit diamond point (MI-53F, Mani, Inc., Japan) with a high-speed airporter handpiece (NSK, Japan) under water coolant until the dentin surface was exposed, which was visually verified using a stereomicroscope at 20X magnification. The ‘test areas’ were delimited to a circle with a dimension of 4 mm in diameter using nail varnish (Lakme, India) at the center of the buccal and palatal surfaces of all teeth. To draw a perfect circle, a modeling wax mold was made with a diameter of 4mm, and then acrylic resin was used to make a button. This same acrylic button was used on all samples for the standardization of all test areas (Figure 1).

The allocated four specimens from each group (1, 2, and 3) for SEM analysis were longitudinally sectioned mesiodistally into buccal and palatal halves using a diamond disc attached to a low-speed micromotor straight handpiece (NSK, Japan). Each halve (buccal and palatal) was further longitudinally sectioned to obtain 2 buccal and 2 palatal halves from each specimen, of which one buccal and one palatal halves were randomly chosen and prepared for a scanning electron microscope (SEM; Sigma 300VP, Carl Zeiss, Oberkochen, Germany) examination to determine the adaptation of dental adhesives to tooth dentin.

All specimens (n = 72) were randomly allocated into three groups, 24 specimens per group. The acrylic blocks of specimens were color-coded for ease of identification. Group 1 (red color): Adper Single Bond 2 (3M ESPE, St. Paul, USA) fifth-generation dental adhesive, Group 2 (blue color): Tetric N-Bond Universal (Ivoclar Vivadent, Liechtenstein) seventh-generation dental adhesive, Group 3 (green color): Prime and Bond Universal (Dentsply Sirona, Switzerland) eighth-generation dental adhesive was used. Each group was further divided into two sub-groups based on diode-laser irradiation and without diode-laser irradiation before photopolymerization of the dental adhesive. The ‘test sites’ on buccal surfaces of teeth were assigned as sub-group 'a' and on palatal surfaces as sub-group 'b'. All the samples were numbered with a marker pen according to their groups and sub-groups a and b.

**Figure 2:**
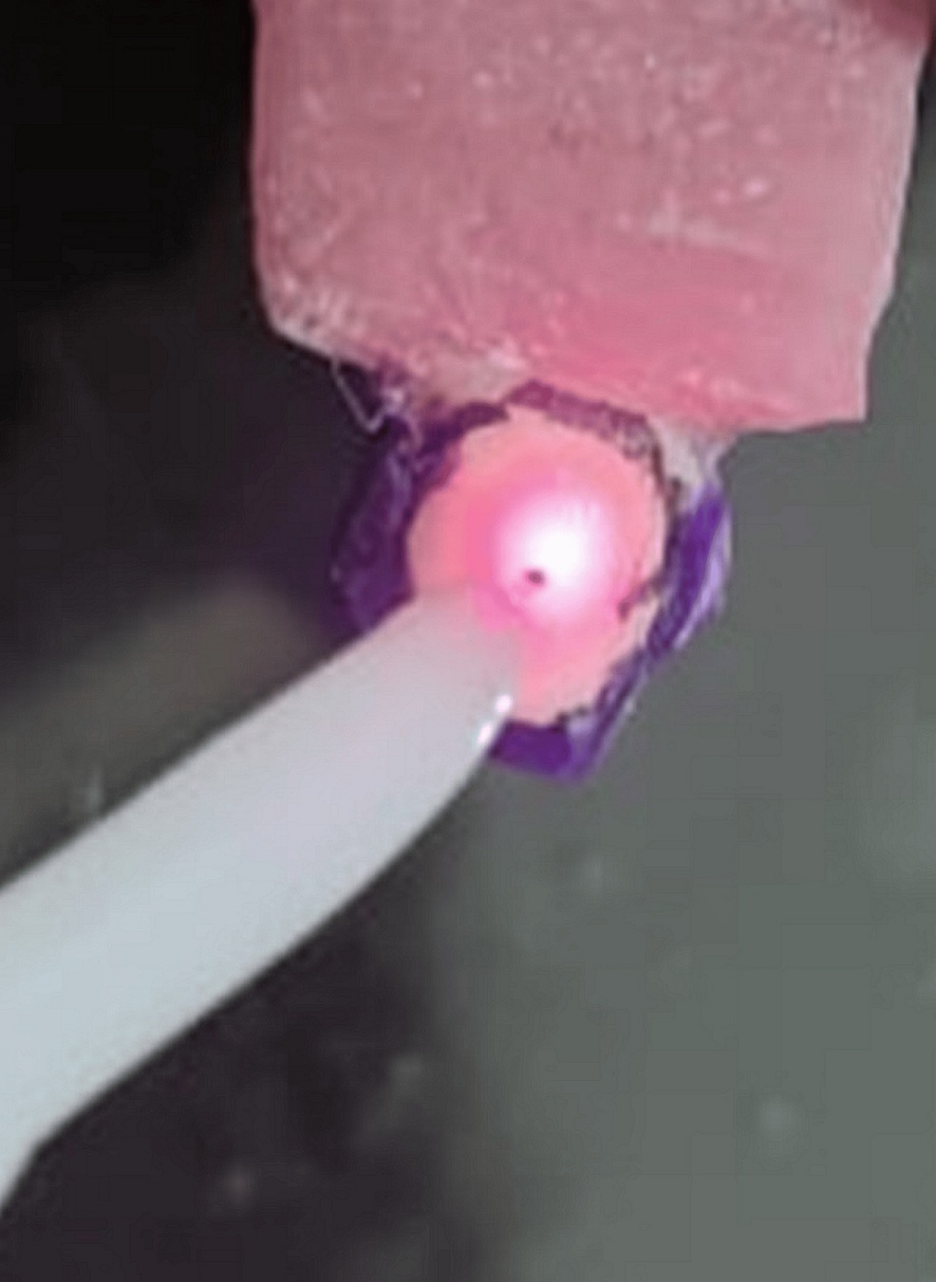
Laser irradiation of the specimen

Group 1 (n = 24): The 4 mm diameter circular windows delimited as ‘test sites’ on buccal and palatal surfaces of teeth were first acid etched using 37% phosphoric acid (Actino Gel, Prevest Denpro, Jammu, India) for 15 seconds, rinsed with water for 10 seconds to completely remove the etchant, and gently dried using an air syringe. Two thin coats of Adper Single Bond 2 dental adhesive were applied and gently rubbed using a microbrush (Reach Global India Pvt Ltd, Pune, India) for 15 seconds, air dried gently for five seconds following manufacturer instructions in ‘Total-etch mode’. Sub-group 1a (buccal surface): Dental adhesive was irradiated with a 940 nm diode laser (Litemedics, Milano, Italy) in continuous wave mode with 0.8 W power, a 400 µm tip diameter and spot size of 0.36 mm^2^, a frequency of 10 Hz, and a total energy of 24 J. It was accomplished in 60 seconds, not in 30 seconds. The diode laser was activated in contact mode, and the full test site was scanned for 30 seconds with the laser tip perpendicularly placed to the tooth surface, ensuring the entire test site was equally irradiated without human error. The laser tip was used in contact mode and was moved from left to right side free handedly on all test areas (Figure 2).

After laser irradiation, the adhesive was then photopolymerized (light-cured) for 10 seconds using a visible light-emitting diode (LED) (Saab, China) dental curing light with a 1 W/cm^2^ power density positioned perpendicular to the tooth surface as closely as possible. Sub-group 1b (palatal surface): Dental adhesive was directly photopolymerized (without prior diode laser irradiation) using an LED dental curing light for 10 seconds with a 1 W/cm^2^ power density positioned perpendicular to the tooth surface as closely as possible.

Group 2 (n = 24) and Group 3 (n = 24) both involved applying dental adhesive to 4 mm diameter circular windows designated as ‘test sites’ on buccal and palatal surfaces of teeth in a thin layer using a micro brush. In Group 2, Tetric N-Bond Universal adhesive was used, while in Group 3, Prime and Bond Universal adhesive was used.

It was slightly agitated for 20 seconds and gently air dried using an air syringe for five seconds to evaporate the solvent until a glossy, uniform layer of adhesive was clinically visible following manufacturer instructions in self-etch mode. Sub-group 3a (buccal surface): Dental adhesive was first irradiated with diode laser followed by photopolymerization using LED dental curing light for 10 seconds, as mentioned above for sub-group 1a. Sub-group 3b (palatal surface): Dental adhesive was directly photopolymerized (without prior diode laser irradiation) using an LED dental curing light for 10 seconds. The light intensity of the LED dental curing unit was verified at regular intervals (every five exposures) with a radiometer (Bluephase meter, Ivoclar Vivadent, U.K.).

Composite restoration placement: After their respective treatment as per groups as mentioned above, hollow disposable plastic straws (Krishna Food, Maharashtra, India) in dimensions of 4 mm × 3 mm (internal diameter of 4 mm, height of 3 mm) were placed on the delimited ‘test sites’ of buccal and palatal surfaces of all teeth as a spacer to standardize the diameter and height of Nano Hybrid Universal composite resin (3M ESPE Filtek Z250 XT, USA) restorations. Composite restorations were done using the incremental layering technique, with each increment in thickness of 1 mm using a Teflon-coated composite carrier instrument (Hu-Friedy, Chicago, USA) and photopolymerized using an LED dental curing light at a high power of 7W/17OO mW/cm^2^ for 20 seconds per increment. The plastic straws were then incised, peeled, and removed, leaving the composite restorations intact, bonded to the tooth dentin, and photopolymerized for an additional 20 seconds with LED curing light to ensure complete polymerization of composite resin. All specimens were then placed in an incubator (Zeal International, New Delhi, India) at 100% relative humidity and 37°C for 24 hours.

To simulate cyclic thermal fluctuations in clinical situations, all specimens were subjected to thermocycling of 2000, two-minute cycles at 4°±2°C and 56°±2°C with a transfer time of five seconds and a dwell time of 30 seconds as per the International Organization for Standardization a thermocycler machine. After thermocycling, four specimens from each group (1, 2, and 3) were randomly allocated for scanning electron microscopy (SEM) analysis to examine the adaptation of dental adhesive to tooth dentin without subjecting these specimens to shear bond strength (SBS) testing, thus preserving the integrity of the adhesive interface. The remaining 60 specimens were then individually subjected to shear bond strength (SBS) analysis in a universal testing machine (Acme Engineers, India) with the knife edge of a chisel rod loaded at the tooth dentin-composite restoration interface with a cross-head speed of 0.5 mm/minute and continued until de-bonding. The SBS values of each specimen (both buccal and palatal surfaces) were expressed in megapascals (MPa) (Newton/mm^2^), dividing the failure load by the bonded surface area [1]. The values were recorded and tabulated.

The de-bonded surfaces of each specimen (buccal and palatal surfaces) were then examined under stereomicroscope at 20X magnification to evaluate the mode of failure at the tooth dentin-composite resin interface. The failure modes were categorized into one of the following [11]. Adhesive (A) is for failures at the adhesive interface with no signs of dentin fracture or remnants of composite resin seen at the test site. Cohesive in Dentin (CD) for failures involving only fractures of dentin seen at the test site. Cohesive in Resin (CR) for failures involving only fractures of composite resin seen at the test site. Mixed (M), when none of the above-mentioned failure modes predominated. The percentage of each type of failure mode was recorded and tabulated.

IBM SPSS (Statistical Package for Social Sciences) software, Version 24 (IBM Corp., Armonk, NY) was used for data analysis. One-way ANOVA (analysis of variance) and the student ‘t’ test are two parametric tests used for statistical analysis to compare the means of more than two groups and any two groups, respectively. A P value ≤ 0.05 was considered statistically significant.

## Results

One-way ANOVA test between the three groups showed that Group 1 (Adper Single Bond 2 fifth-generation dental adhesive in etch-and-rinse mode) and Sub-Group 1b showed the highest shear bond strength (SBS) compared to sub-group 1a, sub-group 2a, and sub-group 3b (Prime and Bond Universal eighth-generation dental adhesive in self-etch mode) used either with or without diode-laser irradiation before photopolymerization. Among the three groups, Group 2 showed the least SBS with or without laser irradiation before photopolymerization. A statistically significant difference was seen in the SBS values expressed in Mpa between the three groups tested (P≤0.05), as summarized in Table 1.

**Table 1:** One-way ANOVA test P: Probability, SD: Standard Deviation Inter-group comparison of Shear bond strength (Mpa) among Group 1, Group 2, and Group 3 specimens with (or) without diode laser irradiation.

Groups	Number of specimens	Sub-group a (with Laser irradiation) Mean ± SD	Sub-group b (without Laser irradiation) Mean ± SD	P-value
Group 1 (fifth generation; Adper Single Bond 2 dental adhesive)	20	13.96 ± 4.43	14.95 ± 4.89	0.50
Group 2 (seventh generation; Tetric N-Bond Universal dental adhesive)	20	10.06 ± 2.84	10.30 ± 3.00	0.80
Group 3 (eighth generation; Prime and Bond Universal dental adhesive)	20	12.03 ± 3.46	10.44 ± 4.09	0.04

A student ‘t’ test was used to compare the sub-groups. A highly significant difference was seen between sub-groups 1a, 2a (P<0.01) and sub-groups 1b, 2b (P<0.01), as shown in Table 2.

**Table 2:** Student ‘t’ test Group 1 vs Group 2 P: Probability, SD: Standard Deviation Student ‘t’ test between sub-groups 1a and 2a, and sub-groups 1b and 2b.

Group 1 vs Group 2	Shear bond strength (Mpa) (Mean ± SD)	P-value
Sub-group 1a	13.96 ± 4.43	0.002
Sub-group 2a	10.06 ± 2.84	
Sub-group 1b	14.95 ± 4.89	0.008
Sub-group 2b	10.30 ± 3.00	

Significant differences were seen between sub-groups 1b and 3b (P<0.05) and no significant differences were seen between sub-groups 1a and 3a (P>0.05) as shown in Table 3, and between sub-groups 2a, 3a (P>0.05) and 2b, 3b (P>0.05) as shown in Table 4. 

**Table 3:** Student ‘t’ test Group 1 vs Group 3 Student ‘t’ test between sub-groups 1a and 3a.

Group 1 vs Group 3	Shear bond strength (Mpa) (Mean ± SD)	P-value
Sub-group 1a	13.96 ± 4.43	0.13
Sub-group 3a	12.03 ± 3.46	
Sub-group 1b	14.95 ± 4.89	0.003
Sub-group 3b	10.44 ± 4.09	

**Table 4:** Student ‘t’ test Group 2 vs Group 3 Student ‘t’ test between sub-groups 2a and 3a, sub-groups 2b and 3b.

Group 2 vs Group 3	Shear bond strength (Mpa) (Mean ± SD)	P-value
Sub-group 2a	10.06 ± 2.84	0.56
Sub-group 3a	12.03 ± 3.46	
Sub-group 2b	10.30 ± 3.00	0.89
Sub-group 3b	10.44 ± 4.09	

In the comparison of failure modes, adhesive failures predominated over the other three types among the specimens. Adhesive failures were predominantly seen in specimens of Group 1, followed by Group 3 and Group 2. On comparison of sub-groups 1a, 2a, and 3a, adhesive failures were predominantly seen in sub-group 3a (75%), followed by sub-groups 1a and 2a. On comparison of sub-groups 1b, 2b, and 3b, adhesive failures were predominantly seen in sub-group 1b (90%) followed by sub-groups 3b and 2b, as summarized in Table 5.

**Table 5:** Percentage of failure modes among three groups A: Adhesive failure, CR: Cohesive resin failure, CD: Cohesive dentin failure, M: Mixed failure.

Groups	Sub-groups	A	CR	CD	M
Group 1	1 a	70%	15%	10%	5%
	1 b	90%	10%	0%	0%
Group 2	2 a	50%	25%	10%	15%
	2 b	70%	15%	5%	10%
Group 3	3 a	75%	20%	5%	0%
	3 b	85%	15%	0%	0%

**Figure 3:**
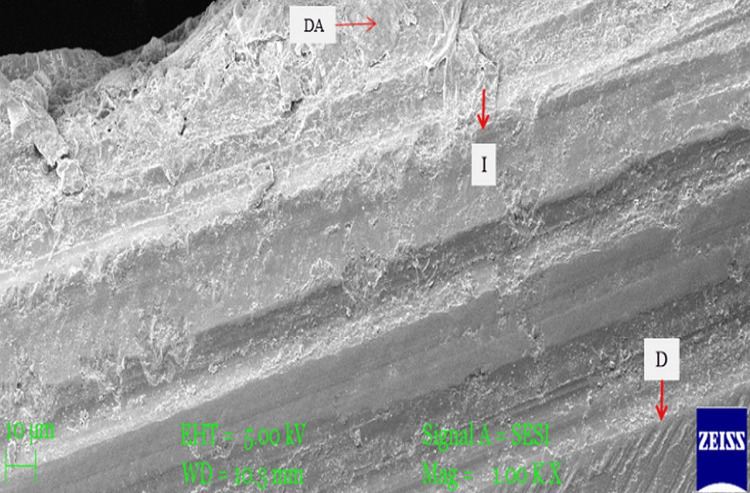
SEM micrograph images of sub-group 1b specimens Scanning electron microscopy (SEM) micrographs of specimens in sub-group 1b (Adper Single Bond 2 etch-and-rinse adhesive) without diode-laser irradiation showed maximum adaptation of adhesive to dentin. DA: Dental adhesive, I: Interface of adhesive-dentin, D: Dentin.

**Figure 4:**
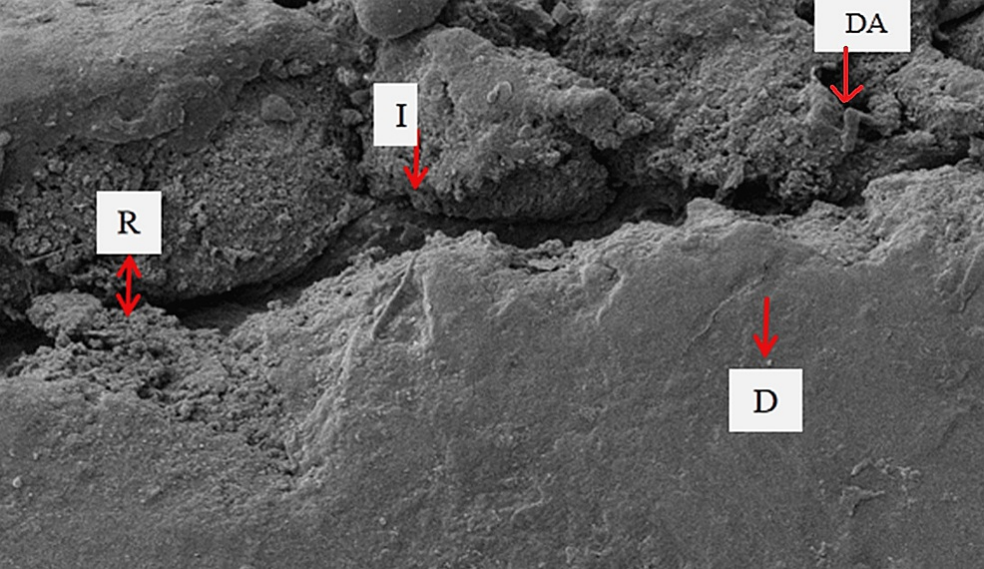
SEM micrograph images of sub-group 2a specimens Scanning electron microscopy (SEM) micrographs of specimens in sub-group 2a (Tetric-N-Bond universal adhesive) with diode-laser irradiation prior to photopolymerization showed very wide separation gaps at the adhesive-dentin interface DA: Dental Adhesive, I: Interface of adhesive-dentin, D: Dentin.

**Figure 5:**
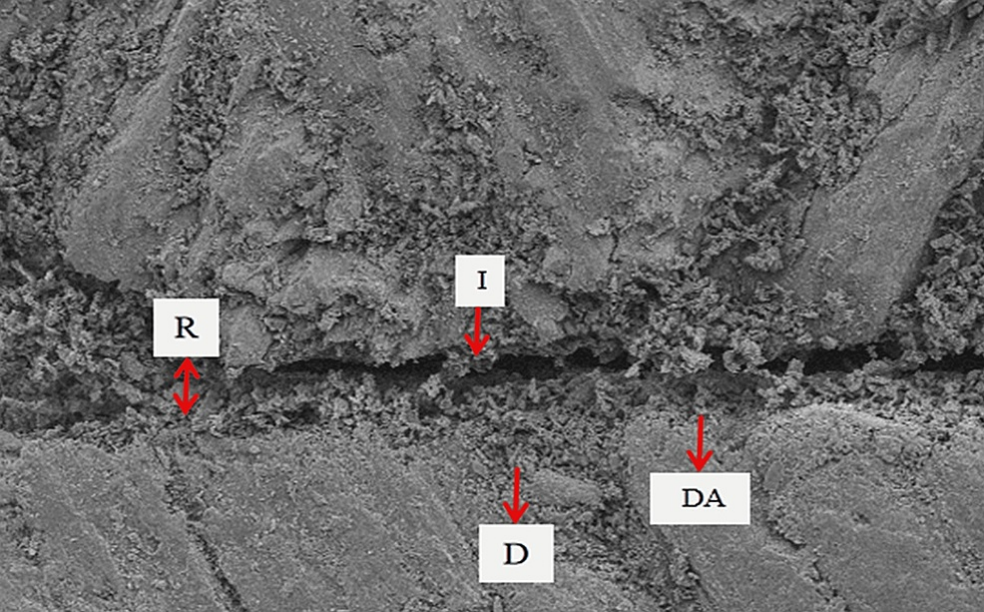
SEM micrograph images of sub-group 2b specimens Scanning electron microscopy (SEM) micrographs of specimens in sub-group 2b (Tetric-N-Bond universal adhesive) without diode-laser irradiation showed wide separation gaps at the adhesive-dentin interface. DA: Dental Adhesive, I: Interface of adhesive-dentin, D: Dentin.

SEM examination of specimens in sub-group 1b showed maximum adaptation of dental adhesive to tooth dentin without any separation gaps; sub-group 1a showed very fine separation at the adhesive-dentin interface; and sub-group 3a showed few separation gaps at the adhesive-dentin interface. However, specimens from sub-groups 2a, 2b, and 3b showed very wide separation gaps, suggesting poor adaptation of dental adhesive to tooth dentin. The specimens of sub-group 1b (Adper Single Bond 2 without diode laser irradiation before photopolymerization) showed the highest SBS (mean 14.95 MPa) and maximum adaptation of dental adhesive to tooth dentin, followed by sub-groups 1a (mean SBS =13.96) and 3a (mean SBS =12.03) (with diode laser irradiation before photopolymerization) showing very fine and fewer separation gaps at the adhesive-dentin interface, respectively (Figures 3-5).

## Discussion

The objective of the shear bond strength test is to determine how strong the bonding of an adhesive system to human dentin is. The shear bond test is one of the most widely used methods to evaluate the bond strength of adhesives to dental hard tissues. According to Alzraikat et al. [12], the shear bond strength test is a simple and quick method to assess the bond strength of dental adhesives. The rationale behind bond strength testing is that the higher the bond strength of an adhesive, the better it can withstand stresses, and the longer the clinical success of composite restoration. The International Organization for Standardization has recommended that in shear bond strength tests, the load should be applied with cross-head speed in the range of 0.45 mm/min to 1.05 mm/min [6]. So, in our study, we employed a cross-head speed of 0.5 mm/minute.

Adper Single Bond 2 is a fifth-generation dental adhesive used in ‘etch-and-rinse’ mode and is composed of ethanol, Bis-GMA, 5 nm silane-treated colloidal silica, 2-hydroxyethyl methacrylate, glycerol-1,3-dimethacrylate, a functional copolymer of polyacrylic and polyitaconic acids, diuretane dimethacrylate, and water [13]. The findings of our study showed Adper Single Bond 2 in etch-and-rinse mode exhibited the highest shear bond strength to tooth dentin, irrespective of whether with or without diode laser irradiation prior to photopolymerization, compared to the use of Tetric N-Bond Universal, Prime, and Bond Universal self-etch adhesives. Hegde et al. [14] study attributed having a separate step of acid etching of tooth dentin using 37% phosphoric acid (etch-and-rinse mode) to deeper penetration of subsequently placed dental adhesive into exposed collagen fibril scaffold with the formation of thicker, longer resin tags compared to self-etch adhesives with shorter resin tag formation, residual acidity, and hydrolytic instability, resulting in poor SBS to tooth dentin.

Our findings showed Adper Single Bond 2 performed on par with the gold standard in SBS values for dentin, and this corroborates the Villela-Rosa et al. [13] study, which demonstrated the highest SBS of Adper Single Bond 2 for human dentin in etch-and-rinse mode compared to other self-etch adhesives used. The findings of our study are in agreement with Knobloch et al. [15]. Raposo and Santana [16] found that the bond strength of total-etch adhesives is higher than that of self-etch adhesives and explained that total-etch adhesives are capable of forming not only a micro-mechanical bond but also a chemical bond with the tooth structure. Soderholm et al. [17] found that etch-and-rinse adhesives performed better in vitro compared to self-etching adhesives, showing higher bond strength values on dentin. Vanajasan et al. [18] concluded in their meta-analysis that one-step self-etch adhesives underperformed compared to conventional three-step adhesives on dentin.

Resaei-Soufi et al. [19] concluded in their study that diode laser irradiation of single bond 2 (etch-and-rinse) adhesive prior to its photopolymerization significantly increased its shear bond strength on human dentin, as irradiation causes a rise in temperature resulting in better evaporation of the residual solvent of the adhesive, thereby enhancing its flow and penetration into the dentin substrate. In our study, Prime and Bond self-etch adhesive (eighth generation) with diode laser irradiation prior to photopolymerization showed higher SBS to dentin, and this can be attributed to laser irradiation further increasing the depth of penetration of already incorporated highly dispersed amorphous silicone-dioxide cetylamine-hydrofluoride particles as nano-fillers with an average particle size of 12 nm in the adhesive. Prime and Bond self-etch adhesive contains a functional acid monomer; 10-MDP (methacryloyloxydecyl dihydrogen phosphate) causes the etching of tooth dentin, allowing other components in the adhesive to penetrate inside the demineralized dentin [20]. 10-MDP can bond chemically with the hydroxylapatite of enamel and dentin, forming 10-MDP calcium salts. In self-etch mode, the remaining hydroxylapatite left over around the collagen scaffold of dentin acts like receptors for chemical reactions, with 10-MDP participating in effective bonding [21].

The findings of our study also corroborate Wiaam et al.'s comparative evaluation that eighth-generation self-etch dental adhesive exhibited maximum SBS, while seventh-generation self-etch dental adhesive registered the least SBS to human dentin either with or without the use of diode laser irradiation before photopolymerization, with the chemical composition of the two adhesive systems directly affecting their bonding capability. Our study findings showed that Tetric-N-Bond Universal seventh-generation dental adhesive exhibited the least SBS to dentin with very wide separation gaps at the adhesive-dentin interface, and this can be attributed to the presence of 2-HydroxyEthyl MethAcrylate (HEMA) in the adhesive and the hydrolytic instability of the methacrylate monomers used. HEMA is a hydrophilic monomer incorporated to prevent phase separation and polymerizes into linear poly-HEMA chains that contain residual water. This water acts as an ionizing medium to allow self-etching, while the ethanol ensures the evaporation of water upon drying. However, it is difficult to evaporate water from these adhesives, and the retained water will rapidly diffuse back from dentin into the adhesive, further enabling it to uptake more water even after light-cured polymerization. The aging process also greatly decreases the bond strength of these adhesives to dentin [22,23].

Thermocycling is a laboratory method used to simulate clinical in-vivo conditions or aging processes with accompanying deteriorating effects like thermal stresses, water absorption, and leakage [24]. So, in our study, all specimens were subjected to thermocycling before their SBS analysis. Gale and Darvell [24] reported that 10,000 thermal cycles arbitrarily correspond to one year of clinical service or aging process, implying that the 2000 thermal cycles chosen in our study roughly represent two months of thermal fluctuations intraorally. However, on the use of thermal cycling among in-vitro studies, Morresi et al. [25], in their literature review, reported no established consensus on a proposed number of thermal cycles to correspond to a specific time in the oral cavity. No standardization was found among the previously published research studies in terms of the power setting of the diode laser used; however, more success was found within the range of 0.75-1 W [9,26]. So, in our study, a diode laser was used with a power setting of 0.8 W.

Study limitation

The limitation of our in-vitro study is that intra-pulpal temperature assessment of diode-lased specimens was necessary to assess their biological safety in order to upgrade this in-vitro study to clinical situations. Hence, future studies should consider this factor.

## Conclusions

Under the conditions of our study, it was found that Adper Single Bond 2 (etch-and-rinse adhesive) without diode-laser irradiation prior to photopolymerization showed the highest SBS with maximum adaptation of dental adhesive to dentin without any gaps or layers of separation at the adhesive-dentin interface, followed by Adper Single Bond 2 adhesive irradiated with diode-laser prior to photopolymerization. Prime and Bond Universal self-etch adhesive with diode-laser irradiation prior to its photopolymerization showed optimum SBS with a very thin layer of separation at the adhesive-dentin interface. Tetric-N-Bond Universal self-etch adhesive with or without the use of diode-laser irradiation showed the least SBS with very wide irregular layers of separation at the adhesive-dentin interface.
